# Glutamate Induces Mitochondrial Dynamic Imbalance and Autophagy Activation: Preventive Effects of Selenium

**DOI:** 10.1371/journal.pone.0039382

**Published:** 2012-06-19

**Authors:** Santosh Kumari, Suresh L. Mehta, P. Andy Li

**Affiliations:** Department of Pharmaceutical Sciences, Biomanufacturing Research Institute and Technology Enterprise, North Carolina Central University, Durham, North Carolina, United States of America; University of Iowa, United States of America

## Abstract

Glutamate-induced cytotoxicity is partially mediated by enhanced oxidative stress. The objectives of the present study are to determine the effects of glutamate on mitochondrial membrane potential, oxygen consumption, mitochondrial dynamics and autophagy regulating factors and to explore the protective effects of selenium against glutamate cytotoxicity in murine neuronal HT22 cells. Our results demonstrated that glutamate resulted in cell death in a dose-dependent manner and supplementation of 100 nM sodium selenite prevented the detrimental effects of glutamate on cell survival. The glutamate induced cytotoxicity was associated with mitochondrial hyperpolarization, increased ROS production and enhanced oxygen consumption. Selenium reversed these alterations. Furthermore, glutamate increased the levels of mitochondrial fission protein markers pDrp1 and Fis1 and caused increase in mitochondrial fragmentation. Selenium corrected the glutamate-caused mitochondrial dynamic imbalance and reduced the number of cells with fragmented mitochondria. Finally, glutamate activated autophagy markers Beclin 1 and LC3-II, while selenium prevented the activation. These results suggest that glutamate targets the mitochondria and selenium supplementation within physiological concentration is capable of preventing the detrimental effects of glutamate on the mitochondria. Therefore, adequate selenium supplementation may be an efficient strategy to prevent the detrimental glutamate toxicity and further studies are warranted to define the therapeutic potentials of selenium in animal disease models and in human.

## Introduction

Glutamate toxicity is a major contributor to neuronal cell death in stroke and other neurodegenerative diseases including Parkinson’s and Alzheimer’s disease [1]. Glutamate-induced cell death is mediated by receptor-initiated excitotoxicity [2] and non-receptor mediated oxidative toxicity [3]. Oxidative glutamate toxicity is initiated by high concentrations of extracellular glutamate that prevent cystine uptake into the cells via the cystine/glutamate antiporter system, resulting in depletion of intracellular cysteine and glutathione [3]. Glutathione depletion induces excessive accumulation of reactive oxygen species (ROS) resulting in oxidative stress. Depletion of antioxidant or excessive accumulation of ROS has detrimental effects on mitochondrial structure and function. Recent studies have demonstrated that oxidative stress may lead to mitochondrial fragmentation thereby altering mitochondrial dynamics [4]. Oxidative stress and mitochondrial dysfunction are considered as primary events in glutamate induced oxytosis [5], although the precise mechanisms are not clear.

Mitochondrial dynamics, i.e. constantly changing in shape, size, and network, is regulated by fission and fusion events, which are controlled by critical regulatory proteins. Among them, dynamin-related GTPase namely Mitofusins 1, 2 (Mfn1, Mfn2) and Optic atrophy 1 (Opa1) control fusion, while dynamin-related protein 1 (Drp1) and Fis1 mediate mitochondrial fission [6]. Mitochondrial fusion regulates calcium buffering capacity, the electron transfer chain (ETC) activity and mitochondrial metabolism [7]. Mitochondrial fission, on contrary, leads to activation of apoptosis, autophagy and neuronal death [8]. The mitochondrial dynamic change can be altered by various factors including ROS production [9].

Autophagy is a mechanism of degradation/recycling of organelles/debris under various stress conditions. Although, autophagy is generally considered to be pro-survival, reports also suggest that many stresses induce cell death via activation of autophagy [10]. Autophagy is mediated in a coordinated process by various proteins such as Beclin 1 and Microtubule-associated protein 1 light chain 3 (LC3). Beclin 1 is part of a Class III PI3K complex that participates in autophagosome formation, mediating the localization of other autophagy proteins to the preautophagosomal membrane [11]. LC3 instead is converted from the cytoplasmic form LC3-I (18 kDa) to the autophagosome-bound form LC3-II (16 kDa) and thus is considered as a marker of autophagy activation [10]. The relationship between glutamate toxicity and mitochondria fragmentation is not known. Likewise, the relationship between glutamate induced autophagy and mitochondrial dynamic change is not clear. However, overexpression of Fis1 or Drp1 has been shown to reduce mitochondrial number through activating mitochondrial autophagy and apoptosis [12], whereas siRNA knockdown of Fis1 or overexpression of a dominant negative isoform of Drp1 (DRP1^K38A^) decreases mitochondrial autophagy [13].

Selenium is a trace element having antioxidants property, and an integral part of many selenium-dependent enzymes such as glutathione peroxidase and thioredoxin reductase [14]. Selenium deficiency is involved in many diseases including muscular dystrophy, endemic fatal cardiomyopathy (Keshan disease), and chronic degenerative diseases [15]; whereas selenium supplementation offers protection in various neurodegenerative diseases [16], [17] by restoring the activity of important antioxidant enzymes and decreasing lipid peroxidation [18]–[20]. Therefore, in the present study we attempted to investigate the potential effects of selenium supplementation on glutamate toxicity. Moreover, efforts were also made to delineate the effect of selenium on mitochondrial dynamics and autophagy in cells exposed to glutamate. To answer these questions, we used murine hippocampal HT22 cells as an *in vitro* model to study the mechanism of selenium protection against glutamate-induced cellular damage. HT22 cells lack functional ionotropic glutamate receptors, therefore, serve as an excellent model of glutamate-induced oxidative neurotoxicity. We found that glutamate exposure damaged HT22 cells, increased ROS production, caused mitochondrial membrane potential hyperpolarization and enhanced oxygen consumption. Glutamate increased the levels of mitochondrial fission markers Drp1 and Fis1, increased percentage of cells with fragmented mitochondria and enhanced autophagy markers Beclin1 and LC-3II. Interestingly, selenium supplementation reduced glutamate-induced ROS production, prevented mitochondrial hyperpolarization, preserved oxygen utilization, maintained mitochondrial dynamic balance and ameliorated autophagy activation, hence showed neuroprotection from glutamate toxicity.

## Results

### Cell Viability

**Figure 1:**
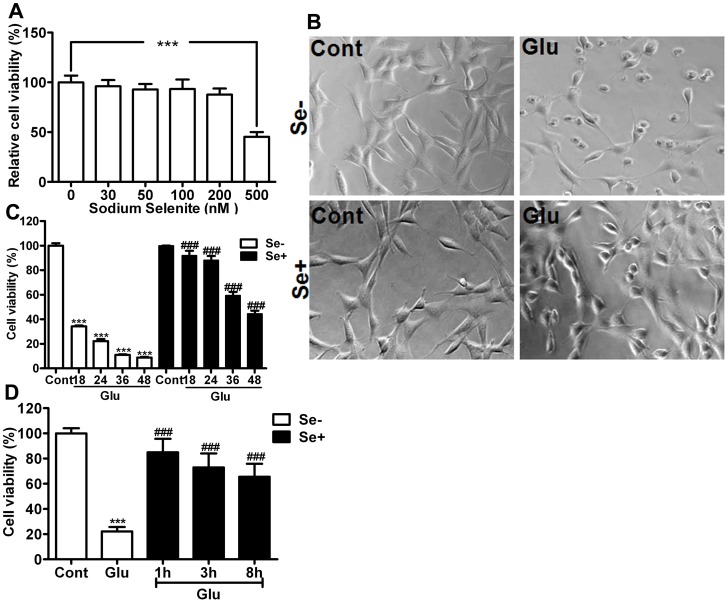
Neuroprotective effect of selenium against glutamate cytotoxicity. (**A**) Concentration-dependent effect of selenium on HT22 cell viability. Selenium was added in the form of sodium selenite (Na_2_SeO_3_) and cell viability was estimated 24 h after addition. The cells tolerated selenium well up to 200 nM, while selenium at 500 nM concentration reduced cell viability to about 50% of control. ***p<0.001 by one-way ANOVA analysis followed by post hoc Scheffe’s test. (**B**) A set of representative microgram of HT22 cells exposed to glutamate (4 mM) with or without selenium (100 nM) for 24 h. Selenium preserved cellular morphologies following glutamate exposure. (**C**) Summarized bar graph showing the effect of selenium on glutamate-induced cell death up to 48 h of glutamate exposure (4 mM). Selenium (100 nM) was added simultaneously with glutamate and the cell viability was measured after 18, 24 26 and 48 h of glutamate exposure using MTT assay. Selenium significantly improved cell survival following glutamate exposure. (**D**) Bar graph shows selenium post-treatment of HT22 cells at 1, 3 and 8 h after glutamate exposure. Protective effect of selenium post-treatment was assessed after 24 h of glutamate exposure. Selenium was able to protect cells from glutamate-induced cell death when applied even 8 h after glutamate exposure. Data were collected from 3 or more independent experiments conducted in triplicate. Values are means±SD and analyzed by two-way ANOVA test followed by post hoc Bonferroni’s test. ***P<0.001 *vs.* control and ###p<0.001 *vs.* non-selenium, glutamate exposed cells. Groups are control (Cont), glutamate exposed (Glu), selenium added (Se+) and non-selenium added (Se-).

To determine the role of selenium on glutamate toxicity-induced neuronal cell death, we simultaneously treated HT22 neuronal cells with glutamate and selenium. Selenium in the form of selenite (Na_2_SeO_3_) up to the 200 nM range was well tolerated by neurons without any influence on cell viability, whereas higher amounts of selenium (500 nM) led to cell death (Fig. 1A). Glutamate treatment resulted in dose-dependent cell death in HT22 cells (data not shown) when measured 24 h after exposure resulting about 80% of mortality at 4 mM concentration. Glutamate (4 mM) induced cell death could be prevented by simultaneous application of selenium (100 nM). Therefore, selenium supplementation significantly (p<0.001) reduced cell death and improved survival of HT22 cells to approximately 90% of control (Fig. 1B and 1C). A time kinetic study revealed that protective effect offered by selenium is not transiently limited to 24 h as the rescue effects were also noticed up to 48 h of glutamate exposure, suggesting the long-term protective effect of selenium. Moreover, experiments with selenium post-treatment revealed that selenium could still be effective to prevent glutamate-induced cell death when given 1–8 h after glutamate exposure (Fig. 1D). The selenium concentration used (100 nM) is reported to be within the physiological range [21].

### Mitochondrial Membrane Potential and ROS Production

**Figure 2:**
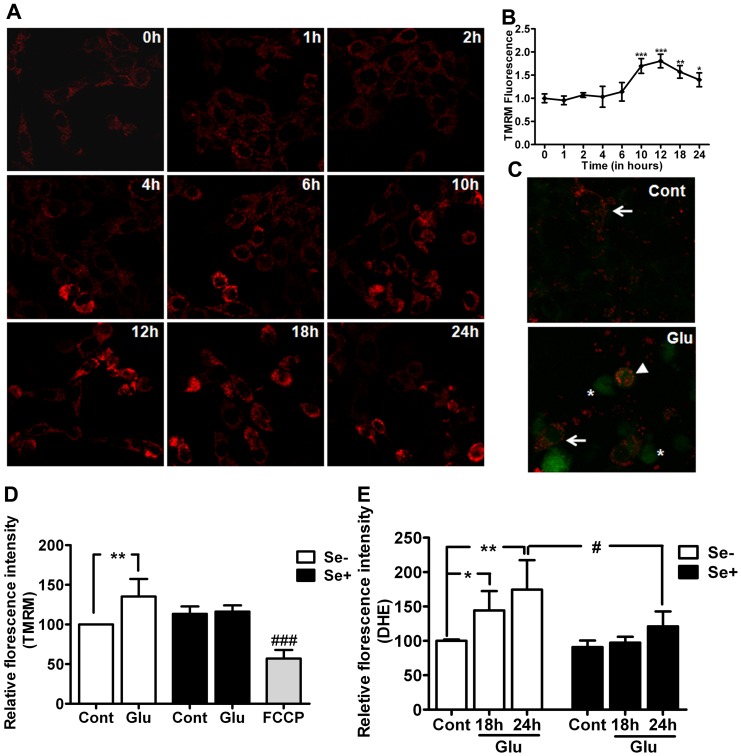
Mitochondrial membrane potential and cellular ROS level in HT22 cells exposed to glutamate (4 mM) and treated simultaneously with or without 100 nM selenium. (**A**) Micrograph of HT22 cells treated with glutamate shows mitochondrial membrane potential change as measured with TMRM. (**B**) Quantitative analysis of TMRM–mitochondrial membrane potential shows that glutamate-induced mitochondrial hyperpolarization, which peaked at 12 h after glutamate exposure. (**C**) Micrograph of mitochondrial membrane potential with JC-1 revealed polarized cells in control whereas glutamate exposure resulted in hyperpolarization and depolarization of cells after 24 h. arrow  =  polarized, arrowhead = hyperpolarized and *(asterisk) = depolarized cells (**D**). Bar graph shows that selenium normalized glutamate-induced change in mitochondrial membrane potential at 24 h of exposure. FCCP was used to dissipate mitochondrial membrane potential and served as a positive control. (**E**) Cellular ROS production measured using DHE. Glutamate significantly increased the production of ROS and selenium successfully prevented the increase in ROS. Data were collected from 5 experiments. Values are means±SD. Two-way ANOVA test followed by post hoc Bonferroni’s test was used for data analysis. *p<0.05, **P<0.01 and ***P<0.001 *vs.* control and #p<0.05 vs. non-selenium treated, glutamate exposed cells. Cont, control, Glu, glutamate, Se-, non-selenium treatment, and Se+, with selenium treatment.

To assess the effects of glutamate on mitochondrial membrane potential, we employed TMRM, a potentiometric fluorescent dye that incorporates into mitochondria in a potential dependent manner. We found gradual change in mitochondrial membrane potential starting at 4 h after glutamate exposure in HT22 cells (Fig. 2A and 2B). Potential peaked at 12 h and thereafter showed recovery but remained elevated as compared to normal level at 24 h of exposure (Fig. 2B). Interestingly, mitochondrial membrane potential seems to be elevated in those cells which were in the early stage of cellular condensation. These results were further confirmed with JC-1 dye (Fig 2C). The results revealed polarized normal cells in control. In contrast, glutamate treatment showed population of normal, hyperpolarized and depolarized cells after 24 h exposure. Hyperpolarized cells appear to be undergoing the process of condensation, which may be an early event during the initiation of cellular damage. Interestingly, selenium supplementation normalized glutamate-induced mitochondrial membrane potential change (Fig 2D).

Previous study has shown that mitochondrial membrane hyperpolarization is associated with ROS production [25], [26]. We subsequently measured superoxide production in the same experimental groups and determined whether the protective effect observed in selenium treatment is associated with its mitochondrial and antioxidative properties. As shown in Fig 2E, superoxide production increased significantly in glutamate exposed cells (p<0.01). Selenium treatment not only prevented glutamate-induced mitochondrial hyperpolarization but also associated with a significantly reduction in ROS production.

### Mitochondrial Oxygen Utilization

**Figure 3:**
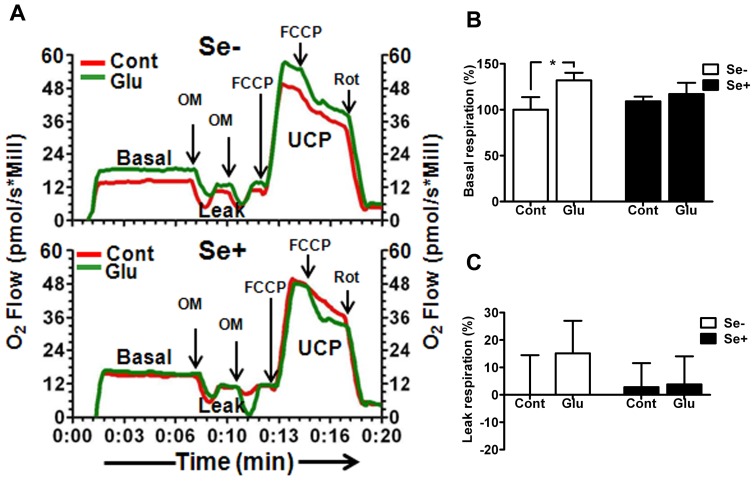
Mitochondrial oxygen utilization in glutamate treated cell with or without simultaneous addition of selenium. (**A**) Oxygraph traces showing respiratory activity of HT22 cells exposed to glutamate (4 mm) with or without 100 nM selenium after 24 h of treatment. Arrows indicate the additions of oligomycin (OM), Carbonylcyanide p-trifluoromethoxyphenylhydrazone (FCCP) and Rotenone (Rot). Oligomycin inhibits ATP synthase and used to reduce respiration to leak state. FCCP was added to stimulate respiration to the uncoupled (UCP) state (mitochondrial respiratory capacity) and rotenone (Rot) to inhibit mitochondrial respiration. (**B**) Calculated basal mitochondrial respiratory rate indicates glutamate increased mitochondrial respiration by close to 30% and selenium normalized the respiratory rate. (**C**) Mitochondrial leak respiratory rate induced by addition of oligomycin. 15 out of the 30% increased respiration after glutamate exposure were not used for ATP production. Selenium supplementation reduced leak respiration in HT22 cells exposed to glutamate. Data were collected from 5 experiments. Values are means±SD. Two-way ANOVA test followed by post hoc Bonferroni’s test was used for data analysis. *p<0.05, *vs.* control. Cont, control, Glu, glutamate, Se-, non-selenium treatment, and Se+, with selenium treatment.

To evaluate the influence of glutamate on mitochondrial function, we measured mitochondrial oxygen utilization using a high-resolution respirometry. The results revealed that glutamate treatment significantly (p<0.01) increased basal cellular respiration in HT22 cell as compared to non-glutamate exposed cells (Fig 3A and 3B). Oligomycin inhibition of ATPase indicated that nearly 15% of oxygen consumed is not used for ATP production and resulted in leak respiration as compared to non-glutamate exposed cells. These results suggest that increased mitochondrial respiration in HT22 cells following glutamate exposure may be accounted for ROS production. Interestingly, simultaneous application of selenium with glutamate reduced ROS production (Fig 2E) and normalized oxygen consumption as well (Fig. 3), in HT22 cells.

### Mitochondrial Dynamic Alteration and Fragmentation

**Figure 4:**
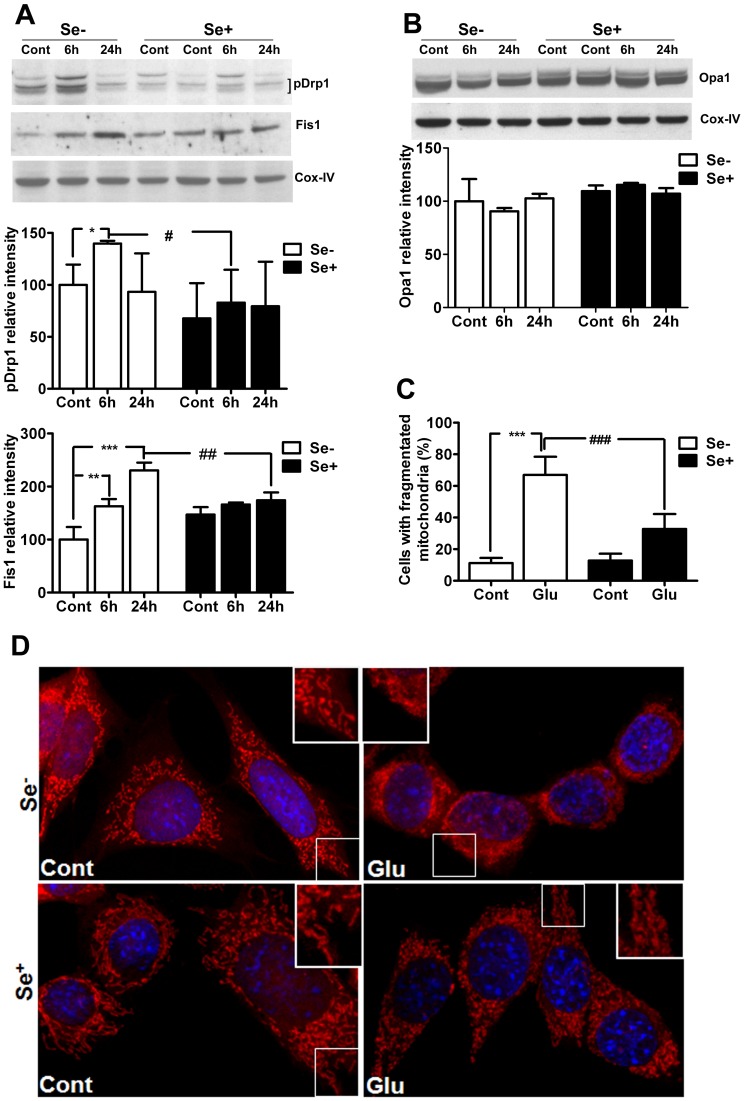
Influences of glutamate and selenium on mitochondrial fission and fusion protein markers. (**A**) Representative Western blots and analysis of mitochondrial fission protein pDrp1 and Fis1. Glutamate increased mitochondrial fission protein markers pDrp1 after 6 h and Fis1 after 6 and 24 h of exposure. Selenium treatment inhibited the corresponding elevation of pDrp1 and Fis1 in these cells. (**B**) Representative Western blot and analysis of mitochondrial fusion protein Opa1. Glutamate did not affect Opa1, nor did the selenium. (**C**) Quantitative analysis of cells with fragmentized mitochondria shows significant increase in number of cells with fragmented mitochondria after glutamate exposure. Simultaneous application of selenium preserved mitochondrial morphology and significantly reduced number of cells with fragmented mitochondria. Minimum of 100 cells per condition were analyzed for mitochondrial morphology. (**D**) Micrograph shows mitochondrial morphology. Mitochondria appear long and thread like in control cells. Glutamate severely affected mitochondrial morphology, leading to mitochondrial fragmentation at 24 h of glutamate exposure. Simultaneous application of selenium prevented glutamate induced mitochondrial fragmentation. Data were collected from 5 experiments. Values are means±SD. Two-way ANOVA test followed by post hoc Bonferroni’s test was used for data analysis. *p<0.05, **P<0.01, ***p<0.001 *vs.* control and #p<0.05, ##p<0.01 *vs.* non-selenium treated, glutamate exposed cells. Cont, control, 6 h, 6 hours after glutamate exposure, 24 h, 24 hours after glutamate exposure, Se-, non-selenium treatment, and Se+, with selenium treatment. Red color indicated mitochondria whereas and blue represents DAPI nuclear staining.

To further determine the influence of glutamate on mitochondrial dynamics we analyzed mitochondrial fission (pDrp1 and Fis1) and fusion (Opa1) regulators, and confirmed the results by viewing mitochondrial morphology using a mitochondrial dye coupled with confocal microscopy. Drp1 is phosphorylated at Ser616 (Cdk1/cyclin B), and Ser637 or 656 (protein kinase A). Phosphorylation of Drp1at Ser616 facilitates mitochondrial fission, whereas its phosphorylation at Ser637 or Ser656 inhibits it [27], [28]. Therefore, in the present study, we used antibody which detect Drp1 phosphorylated at Ser616. As shown in Fig. 4A & 4B, pDrp1 protein level increased after 6 h and returned to control level after 24 h of glutamate exposure. Fis1 levels increased after 6 h and further elevated after 24 h of glutamate exposure as compared to non-glutamate exposed control. In contrast, fusion protein Opa1 was not changed by glutamate exposure (Fig 4B). Interestingly, selenium supplementation prevented glutamate-induced increases in pDrp1 and Fis1 after 6 and/or 24 h of exposure.

To further determine the role of glutamate in mitochondrial fragmentation and preservation of mitochondria structure by selenium supplementation, we analyzed mitochondrial morphology with MitoTracker® Red CMXRos dye. Cell images were captured with confocal microscope (Fig 4D). Result revealed two types of mitochondrial morphologies, tubular and fragmented. Tubular structure of mitochondria was prominent in control cells whereas glutamate exposure severely reduced mitochondrial size and disrupted mitochondrial reticularity. Quantitative analysis of cells with mitochondrial morphologies revealed that number of cells with fragmented mitochondria increased significantly following glutamate exposure as compared to control (Fig. 4C). Selenium supplementation in control *per se* seems to increase mitochondrial tubularity and also preserved mitochondrial tubular structure from glutamate exposure. Therefore, selenium supplementation significantly reduced the number of cells with fragmented mitochondria. These results indicate that glutamate tilts the mitochondrial dynamic balance toward fission, whereas selenium supplementation restores the mitochondrial dynamic balance.

### Autophagy Activation

**Figure 5:**
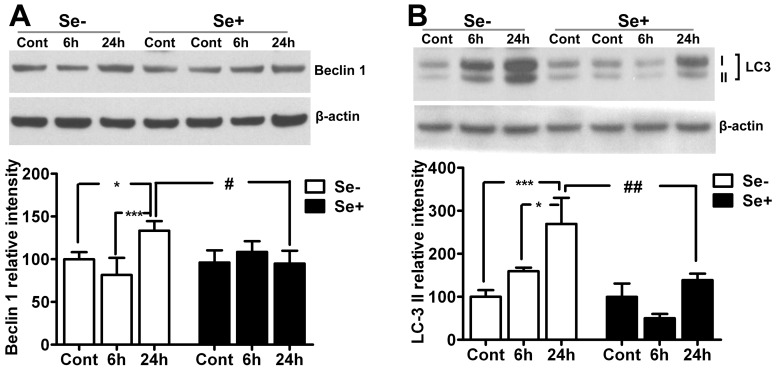
Influences of glutamate and selenium on autophagy protein markers Beclin 1 and LC3-II. Glutamate caused increased of Beclin 1 and LC3-II after 24 h of exposure. Selenium supplementation inhibited the glutamate-induced activation of autophagy. Data were collected from 5 experiments. Values are means±SD. Two-way ANOVA test followed by post hoc Bonferroni’s test was used for data analysis. *p<0.05, ***p<0.001 *vs.* control and #p<0.05, ##p<0.001 *vs.* non-selenium treated, glutamate exposed cells. Cont, control, 6 h, 6 hours after glutamate exposure, 24 h, 24 hours after glutamate exposure, Se-, non-selenium treatment, and Se+, with selenium treatment.

Because glutamate has been shown to activate autophagy in a concentration-dependent manner in the HT22 cells [10], we decided to investigate whether prevention of glutamate cytotoxicity by selenium involves inhibition of autophagy activation. To that end, protein levels of Beclin 1 and conversion of LC3-I to LC3-II were determined using Western blotting. As shown in Fig. 5, glutamate exposure significantly (p<0.05) increased Beclin 1 levels at 24 h in HT22 cells as compared to control. Interestingly, selenium supplementation prevented the elevation of Beclin 1 at 24 h of glutamate exposure. Therefore, Beclin 1 levels significantly (p<0.05) decreased in selenium treated HT22 cells after 24 h of glutamate exposure as compared to that of the glutamate exposed cells.

**Figure 6:**
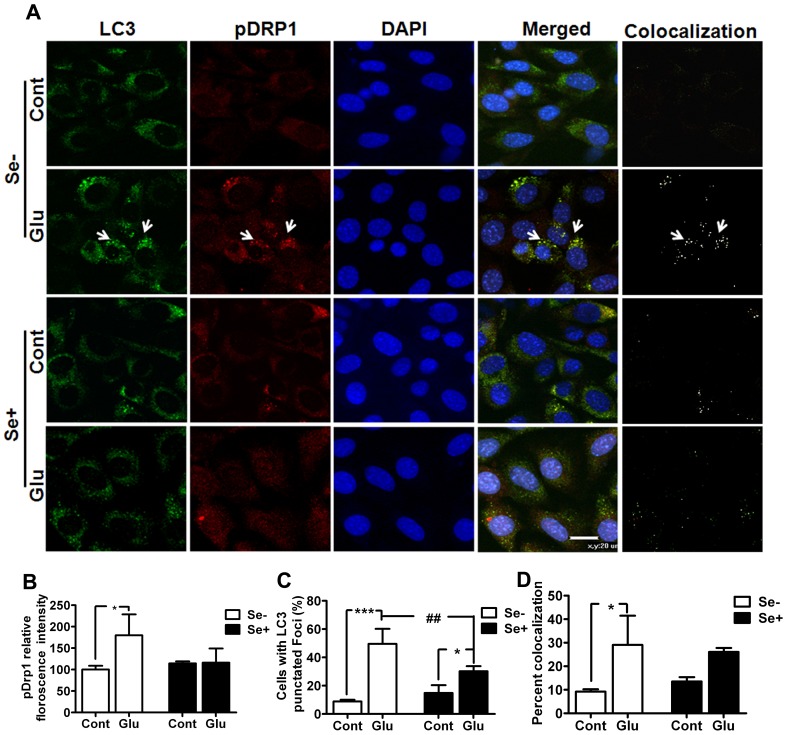
Co-immunolocalization of LC3 and pDrp1 after glutamate exposure with or without selenium. (**A**) Double immunofluorescent staining revealed accumulation of LC3 punctuated foci in cells exposed to glutamate. The aggregations of LC3 co-localized (white punctate foci) with pDrp1 as revealed by analysis with ImageJ Colocalization Plugin. Since pDrp1 locates in the mitochondria and LC3 locates in autophagosomes, the colocalization of these two suggests the activation of mitochondrial autophagy. Selenium supplementation reduced the aggregation of LC3 and pDrp1. (**B**) Fluorescent intensity of pDrp1 in cells treated simultaneously with glutamate and selenium. (**C**) The percentage of cells with punctuated LC3 foci. Glutamate exposure significantly increased the number of cells with punctated LC3 foci, whereas selenium supplementation prevented the increase in LC3 punctation. (D) Co-localization of pDrp1 and LC3 shows significant number of cells following glutamate treatment. Selenium treatment, in contrast showed tendency to lower the number of cells with co-localized signal as compared to glutamate exposure alone. Data was collected from more than two independent experiments. Values are means±SD. Two-way ANOVA test followed by post hoc Bonferroni’s test was used for data analysis. *p<0.05, ***p<0.001 *vs.* control and ##p<0.001 *vs.* non-selenium treated, glutamate exposed cells. Green color, LC3 staining, red, pDrp1 staining, blue, DAPI nuclear staining and yellow/white, colocalization of LC3 and pDrp1. Cont, control, Glu, glutamate, Se-, non-selenium treatment, and Se+, with selenium treatment.

Similar trend was also observed with LC3, where LC3-I to II conversion was increased at 24 h of glutamate exposure. Thus, LC3-II levels was significantly (p<0.001) higher at 24 h of glutamate exposure when compared with control. In contrast, selenium supplementation prevented (p<0.01) the conversion of LC3-I to LC3-II at 24 h. These results were further confirmed with immunofluorescence studies. As shown in Fig. 6A, the formation of punctated LC3 staining indicate the conversion of LC3 (form I to II) and its recruitment into autophagosomes. The punctated LC3 staining increased in HT22 cells exposed to 24 h of glutamate (6A&C). These staining patterns overlapped with pDrp1 staining, implying that fragmented mitochondria may be cleared through autophagy dependent mechanism. Interestingly, selenium supplementation not only reduced the number of cells with punctated LC3 foci (Fig 5 and 6A&C) but also decreased pDrp1 (Fig. 4 and 6B) level in HT22 cells exposed to glutamate. Therefore, the staining pattern of these proteins decreased following simultaneous application of selenium and glutamate (Fig. 6A). ImageJ co-localization analysis of pDrp1 and LC3 punctation revealed that glutamate significantly increased the number of cells with co-localized pDrp1 and LC3 signal (Fig. 6D), whereas selenium treatment showed tendency to lower the number of cells with co-localized signal following glutamate exposure compared to glutamate treatment alone. These results thus may imply that selenium prevent glutamate cytotoxicity by preventing mitochondrial fragmentation and subsequent autophagy activation.

## Discussion

The present study demonstrates that glutamate toxicity is linked to mitochondrial membrane hyperpolarization, increased production of ROS, elevated mitochondrial oxygen consumption, disturbance of mitochondrial dynamic balance towards fission and activation of autophagy. The preventive effects of selenium against glutamate toxicity is associated with stabilization of mitochondrial membrane potential, reduction of ROS production, restoration of mitochondrial dynamics and prevention of autophagy activation.

Our present study revealed that selenium mediated neuroprotection was not limited to early phase (24 h) of glutamate-induced cell death, since the protective effect was still observed when study extended to 48 h following glutamate exposure. Interestingly, selenium was also able to protect cells form glutamate-induced cell death when applied hours after glutamate exposure, which clearly indicates the preventive and putative therapeutic nature of selenium [29].

Selenium may exert its neuroprotection through two mechanisms. First, it is generally believed that selenium exerts its biological effects through incorporation into selenoproteins as selenocystein. Many of the selenoproteins function as antioxidant enzymes. For examples, selenium increases glutathione peroxidase activity, the latter catalyzes hydroperoxide to water and carbon dioxide. However, a study conducted by Savaskan et al [29] also showed that selenate, which lacks direct antioxidative properties, had nearly 75% protection as compared to nearly 100% cell viability with selenite after glutamate exposure [29], suggesting that selenite prevents glutamate-induced cell death directly or indirectly by lowering ROS production. Secondly, selenium has been shown to activate PI3K/Akt pathway [30], which regulates many downstream signaling pathways including cell proliferation and metabolism. In a parallel study conducted in our lab, we observed that selenite induced phosphorylation of Akt, PKA and CREB and increased protein levels of peroxisome proliferator-activated receptor (PPAR) gamma-1α (PGC-1α) and nuclear respiratory factor 1 (NRF1) [Mehta, Mendelev, Kumari, Li unpublished data], further supporting the hypothesis that selenium modulate transcription factors.

Glutamate induces cell death through receptor-initiated excitotoxicity or non-receptor mediated oxidative toxicity [2], [3]. In the present study, we observed a significantly increased superoxide level following glutamate treatment, whereas selenium supplementation prevented the glutamate-induced superoxide production. Previous published data have shown that selenium reduces both superoxide and hydroperoxide after various injuries [21], [29], [30] The neuroprotection offered by selenium thus seems to be partially associated with lowering ROS production, since ROS is linked to necrosis at early hour (12 h) and apoptosis predominantly at late hour (12–24 h) of glutamate exposure [5], [31], [32].

The increased production of ROS following glutamate exposure may be associated with the elevation of mitochondrial membrane potential observed in the present study. Mitochondrial membrane potential gradually increased, peaked at 12 h and reduced thereafter but remained elevated 30% above control level after 24 h of glutamate exposure in TMRM imaging study that was confirmed by JC-1 assay. Three populations of cells were observed after 24 h of glutamate exposure: polarized intact cells, depolarized cells and hyperpolarized cells. Interestingly, hyperpolarized cells appeared to be undergoing early stage condensation. Thus increased mitochondrial membrane potential may have increased oxygen consumption and ROS production in glutamate-treated as compared to non-glutamate treated [25], [26]. Likewise, mitochondrial hyperpolarization can trigger mitochondrial fragmentation [33] and contribute to increased oxygen consumption and increased electron flow [34]. Although mitochondrial oxygen consumption was increased by glutamate, such increase did not fully account for ATP production. In fact, about 50% (15% out of 30%) of the consumed oxygen was not used for ATP production based on the experiments using oligomycin to inhibit mitochondrial complex V, which is responsible for ATP production. It is not unlikely that the increased oxygen consumption was used for ROS generation.

Selenium provides beneficial effects under various stress condition by regulating oxidative stress and mitochondrial dysfunction [16]–[21], [28], [35], [36]. In the present study, we have shown that supplementation of selenium in the form of sodium selenite within physiological range ameliorated glutamate-induced cell death. Interestingly, selenium reduced the glutamate-induced mitochondrial hyperpolarization and ROS production and normalized mitochondrial oxygen consumption. Previous studies have shown that selenium is capable of restoring the activity of important antioxidant enzymes such as glutathione peroxidases and thioredoxin reductase [18], [20], [21]. Thus, it is tempting to speculate that glutamate disturbs cellular redox status of the cell, which may be responsible for some of the major biological consequences. Selenium, in contrast, protects cells from glutamate toxicity by maintaining the cellular redox condition. In addition, we have observed that selenium prevented the deterioration of mitochondrial respiratory complex enzyme I, II+III and IV induced by hypoxia [Mehta, Kumari, Mendelev, Li, unpublished data].

Emerging evidence suggests that mitochondria are targets of ROS and ROS production may result in mitochondrial fragmentation through differential modulation of mitochondrial fission-fusion proteins [4]. Presently, we observed Ser616 phosphorylation of Drp1 and Fis1 levels were increased following glutamate exposure. It has been reported that overexpression of Drp1 leads to mitochondrial fragmentation whereas dominant negative Drp1 slowed the rate of mitochondrial fragmentation and decreased total cell death [37], [38]. In a recent study published by Grohm and colleagues, inhibition of mitochondrial fission by Drp1 small interference RNA or small molecular inhibitors protected neurons against glutamate toxicity and hypoxia in vitro and reduced infarct volume after cerebral focal ischemia in vivo [39]. The mitochondrial morphology observed through mitochondrial imaging study support the fission marker results. Thus, normal intact control cells exhibited reticular mitochondrial network, whereas glutamate reduced the reticularity of the mitochondria, suggesting mitochondrial fragmentation. These results suggest that glutamate affects mitochondrial dynamics which favors mitochondrial fission. Our results support the notion that glutamate may affect mitochondria beyond the bioenergetics and ROS generation [40].

One consequence of mitochondrial fission is mitochondrial fragmentation and condensation [40], which is clearly evidenced in our study. It is known that mitochondrial fragmentation may activate mitochondrial autophagy. To study whether autophagy is activated following glutamate exposure, we measured Beclin 1 and LC3-II levels that have been commonly used as markers for autophagy activation [41]. Our results indicated the activation of autophagy following glutamate exposure, as reflected by increased levels of Beclin1 and conversion of LC3-I to LC3-II. Moreover, we observed the co-immunolocalization of pDrp1 and LC3. Since pDrp1 locates on mitochondrial membrane and LC3-II locates in autophagosome, the colocalization of the two suggests the association of autophagy with mitochondrial fission. As we have mentioned previously, overexpression of Fis1 or Drp1 reduces mitochondrial number by activating mitochondrial autophagy and apoptosis [12], while on the contrary, siRNA knockdown of Fis1 or overexpression of a dominant negative isoform of Drp1 decreases mitochondrial autophagy [13].

The influences of selenium on mitochondrial fission/fusion and autophagy have not been studied before. In the present study we also demonstrated that selenium restored the imbalance between mitochondrial fission and fusion and prevented the activation of autophagy induced by glutamate. It is likely that reduced fission regulators would be associated with inhibition of glutamate-induced mitochondrial fragmentation and cell death. Activation of autophagy is a double edged sword. On one hand, physiological activation of autophagy removes and recycles damaged cellular and subcellular components and denatured proteins, which maintains a health environment for cell well being. On the other hand, excessive activation of autophagy may results in cell death and clearance. It is possible that glutamate-induced autophagy activation may be related to cell death and selenium reduces glutamate-induced cell death by inhibiting the activation of autophagy.

Taken together, glutamate at a concentration sufficient to induce cell injury targets mitochondria as reflected by altered mitochondrial membrane potential, mitochondrial oxygen consumption and mitochondrial dynamics, which are associated with increased production of ROS and activation of autophagy. These unappreciated effects of glutamate are prevented by selenium supplementation. Therefore, selenium reduces glutamate-induced ROS production, stabilizes mitochondrial potential, normalizes oxygen utilization, restores mitochondrial dynamic balance and prevents autophagy activation. Adequate selenium supplementation is an efficient strategy to prevent the detrimental glutamate toxicity and further study should be conducted to explore its therapeutic potential in animal models and in human.

## Materials and Methods

### Cell Culture Maintenance, Experimental Treatments and Harvesting

Mouse hippocampal HT22 cells were kindly provided by Dr. Panee [21]. HT22 cells were cultured in Dulbecco’s Modified Eagle Medium (DMEM)/F12 containing 10% fetal bovine serum (FBS), 2 mM glutamine, and 200 mM streptomycin/penicillin (Invitrogen) and maintained at 90%–95% relative humidity in 5% CO_2_ at 37°C. The culture medium was renewed every 3 days. Varying concentration of glutamate (1–5 mM) and sodium selenite (50–500 nm) were tested and evaluated for cell viability after 24 h of treatment.

### Cell Viability

For determining cell viability, the 3-(4,5-dimethylthiazol-2-yl)-2,5-diphenyltetrazolium bromide (MTT) assay was used. HT22 cells were seeded in 96-well plates at a density of 10,000 cells per well. Glutamate stock solution was diluted in DMEM culture medium immediately before treatment. Similarly, cells were also incubated with 100 nM sodium selenite with or without glutamate. After 24 h of treatment, cells in each well were incubated with 10 µl MTT (5 mg/ml) at a final concentration of 500 µg/ml for 1 h. After incubation, the medium was removed from the wells and formazan formed was dissolved in dimethyl sulfoxide (DMSO, 100 µl). The absorbance was read with a dual-monochromator, multi-detection microplate reader (SpectraMax M5 Molecular Device, Sunnyvale, CA) at 540 nm. Cell viability was expressed as a percentage of the control.

### Determination of Mitochondrial Membrane Potential and ROS Levels

Mitochondrial membrane potential was measured using the tetramethylrhodamine methyl ester (TMRM). TMRM is a cell-permeant, cationic fluorescent dye that is readily sequestered by active mitochondria. Briefly HT22 cells were incubated with 30 nM dye at 37°C for 1 h. Cells were washed twice in PBS and fluorescence was measured (excitation: 530 nm and emission: 573 nm) with a Fluoromax-4 spectroflorometer (HORIBA Jobin Yvon Inc, Edison, NJ). Carbonylcyanide p-trifluoromethoxyphenylhydrazone (FCCP 5 µM) was used as a positive control to dissipate mitochondrial potential. For live imaging, cells were grown on slides and incubated with either 100 nM TMRM or JC-1 dye (5 µg/ml). JC-1 was excited at 488 nm and emission of JC-1 monomer (depolarized) and J-aggregate (polarized mitochondria) forms were captured at 525/50 and 605/75 nm, respectively. The concentration of TMRM was maintained in the bathing solutions throughout the experiments [22]. Images were captured with Nikon laser-scanning confocal microscope at fixed time intervals. To avoid the photobleaching, laser power was attenuated to the minimal level. Intensity measurement was performed using NIH Image J software. Minimum of 20 cells per microscopic field was analyzed to determine the changes in mitochondrial membrane potential.

Intracellular superoxide anion production was determined using dihydroethidium (DHE) as a fluorescent probe. Briefly, cells (2×10^6^/ml) were incubated with the DHE (2.5 µM) for 30 min at 37°C, after which they were washed, resuspended in phosphate buffered saline (PBS) and analyzed for fluorescence intensity using Fluoromax-4 spectroflorometer (HORIBA Jobin Yvon Inc, Edison, NJ) at the excitation and emission wavelengths of 480 nm and 590 nm respectively. The florescence recorded was represented as relative fluorescence intensity (RFI).

### Mitochondrial Oxygen Consumption Measurement

Oxygen consumption was measured with high-resolution respirometry (Oxygraph, Oroboros Instruments, Innsbruck, Austria) in a glass chamber containing 2 ml of media (DMEM/F12 with 10% FBS) at 37°C. The medium containing 5×10^6^ cells was equilibrated in ambient room air with continuous stirring (750 rpm) for 10 min. The chamber was closed to start recording the oxygen consumption at 2 second intervals and the recording was stopped after stabilization of the O_2_ consumption signal. Oligomycin (2 µg/ml) was used to inhibit ATP synthase and to determine the leak respiration. Subsequently, carbonylcyanide-4- (trifluoromethoxy) -phenylhydrazone (FCCP 0.5 µM steps) was titrated to uncouple respiration and to determine mitochondrial functional capacity. Respiration was finally inhibited with rotenone (0.5 µM). The difference in oxygen consumption between HT22 cells treated with glutamate in the presence or absence of selenium was calculated using DataGraph software (Oroboros Instruments). Respiration rates were computed and data were transformed into percent of respiration rate of control.

### Subcellular Fractions

Mitochondrial and cytosolic fraction was used in the study and the fractions were isolated as described previously with minor modifications [23]. Briefly, cells were washed with PBS and resuspended in cytosolic lysis buffer (250 mM sucrose, 70 mM KCl, 137 mM NaCl, 4.3 mM Na_2_HPO_4_, 1.4 mM KH_2_PO_4_ pH 7.2, 200 µg/ml digitonin, 100 mM PMSF, protease inhibitor cocktail) for 5 minutes on ice. These cells were centrifuged at 1,000× *g* for 5 minutes to separate the supernatant as a cytosolic fraction and the pellet was resuspended in two volumes of mitochondrial lysis buffer (50 mM Tris-HCl pH 7.4, 150 mM NaCl, 2 mM EDTA, 2 mM EGTA, 0.2% (v/v) Triton X-100, 0.3% NP-40, PMSF, protease inhibitor cocktail) for 5 minutes on ice. The resulting suspension was centrifuged at 10,000× *g* for 10 minutes and the supernatant was collected as the mitochondrial fraction. The purity of the fractions has been previously verified [24]. Protein concentration was determined using the Bradford assay.

### Western Blotting

Western blotting analysis was used to determine the level of specific proteins in the subcellular fractions. Briefly, same amount of protein (20 µg) was loaded in each lane of a 4–12% NuPAGE gel (Invitrogen). Following electrophoresis, proteins were transferred to a nitrocellulose membrane (Invitrogen) and blocked for nonspecific binding with 5% skimmed milk. The membrane was incubated for target proteins using primary antibodies specific for phospho-Drp1 (Ser616 1:800, 3455, Cell Signaling USA), Fis1 (1∶500 sc-98900, Santa Cruz Biotechnology, CA, USA), Opa1 (1∶1000 sc-30572, Santa Cruz Biotechnology, CA, USA), Beclin 1(1∶500 (sc-11427, Santa Cruz Biotechnology, CA, USA), LC-3 (5 µg/ml, M115-3, MBL MA, USA), COX IV (1∶1000 (ab14744, Abcam Inc, MA, USA) or β-actin of 1∶1000 (A1978, Sigma) overnight at 4°C. The membranes were incubated with horseradish peroxidase-conjugated secondary antibodies for 1 h at room temperature. The blots were then developed using the Supersignal West Dura Extended Duration Substrate (Thermo Scientific). The β-actin or COX-IV bands were used as internal loading controls and the ratios of the targeted proteins and loading control were calculated and presented as final results.

### Mitochondrial Staining

Cells were grown on Lab-Tek II Chamber Slide (Thermoscientific) and labeled with MitoTracker®Red CM-XRos (Invitrogen) at 37°C in a humidified 5% CO_2_ atmosphere for 30 min. The cells were then fixed with paraformaldehyde in culture medium for 15 min at room temperature. After washing with PBS twice, cells were mounted with Vectashield mounting medium containing DAPI (Vector Laboratories) and analyzed by Nikon laser-scanning confocal microscope at 600X final magnification.

### Immunofluorescent Staining

HT22 cells were grown on Lab-Tek II Chamber Slide (Thermoscientific). The cells were fixed with 4% paraformaldehyde for 20 min, washed with PBS and permeabilized in Triton X-100 (0.3%) for 5 min. The cells were blocked with 10% donkey serum for 1 h at room temperature. After blocking, cells were incubated overnight at 4°C with primary antibodies against LC3 (1∶1000, Abcam Inc, Cambridge MA), and pDrp1 (1∶1600, Cell Signaling Technology, Danvers, MA). Cells were washed and incubated for 2 h at room temperature with secondary antibodies conjugated with Alexa Fluor 488 or 568 (1∶500 dilution; Invitrogen). Finally, slides were mounted with Vectashield mounting medium containing DAPI (Vector Laboratories). Slides were scanned with Nikon laser-scanning confocal microscope at 400X final magnification. Minimum three fields per group were captured. The number of cells with LC3 punctate staining was counted and the fluorescent intensity of pDrp1 as well as co-localization of pDrp1 and LC3 was analyzed using NIH ImageJ with colocalization plugin.

### Statistical Analysis

All data are presented as means ± SD. Multiple comparisons were performed with one-way ANOVA followed by post hoc Scheffe’s test or two-way ANOVA followed by Bonferoni test whenever appropriate. p<0.05 is considered statistically significant.
